# Maladie du Sommeil: Une Cause de Faux Positifs des Tests de Diagnostic Rapide du VIH

**DOI:** 10.48327/mtsibulletin.2021.115

**Published:** 2021-06-20

**Authors:** J. Bonnet, S. Ducroix-Roubertou, S. Rogez, D. Ajzenberg, B. Courtioux, J.-F. Faucher

**Affiliations:** 1 Université de Limoges, U1094, Neuroépidémiologie tropicale, Institut d'épidémiologie et de neurologie tropicale, GEIST, Limoges, France.; 2 Service des maladies infectieuses et tropicales, Centre hospitalier universitaire de Limoges, Limoges, France; 3 Service de bacte?riologie, virologie, hygie?ne, Centre hospitalier universitaire de Limoges, Limoges, France; 4 Service de parasitologie-mycologie, Centre hospitalier universitaire de Limoges, Limoges, France

**Keywords:** Trypanosomose humaine africaine, Test de diagnostic rapide VIH, Faux positifs, Limoges, France, Uíge, Bengo, Kwanza Norte, Angola, Human African Trypanosomiasis, HIV rapid diagnostic test, False positives, Hospital, Limoges, France, Uíge, Bengo, Kwanza Norte, Angola

## Abstract

L'approche des mécanismes liés aux résultats faussement positifs des tests de diagnostic rapide (TDR) du VIH chez les patients atteints de la trypanosomose humaine africaine (THA) ou maladie du sommeil contribuera à améliorer la précision du dépistage de l'infection par le VIH, dans les zones endémiques pour la THA.

Nous présentons le cas d'une patiente du Congo, prise en charge pour une méningoencéphalite opportuniste d'un sida lors de son admission sur la base d'un TDR VIH qui s'est ensuite révélé faussement positif, et dont le diagnostic était celui de maladie du sommeil. Une étude de cohorte rétrospective réalisée ultérieurement chez des patients atteints de THA, montre que la plupart des TDR VIH positifs avant traitement sont des faux positifs et que la moitié d'entre eux ont disparu à la fin du traitement, ce qui suggère une clearance rapide d'anticorps en réaction croisée avec le parasite.

Une part importante des TDR VIH faussement positifs disparaît avant la fin du traitement trypanocide. Là où les tests sérologiques Elisa VIH ne sont pas rapidement accessibles, le renouvellement du TDR VIH en fin de traitement de la THA permet d'identifier environ la moitié des TDR VIH faussement positifs.

## Abstract (en)

Approaching the mechanisms related to false positives HIV rapid diagnostic tests (RDT) in patients with sleeping sickness may help to improve the accuracy of screening for HIV infection in areas endemic for Human African trypanosomiasis (HAT).

We report on a patient from Congo who was managed like an AIDS-associated meningoencephalitis, based on a false positive HIV RDT at admission, and eventually received a diagnosis of sleeping sickness. A further retrospective cohort study performed in patients with HAT shows that most of positive HIV RDT obtained prior to treatment for sleeping sickness are false positives. We found that half of them were cleared at the end of treatment course, suggesting an early clearance of some antibodies involved in cross-reactivity.

A substantial clearance of HIV RDT false positives occurs during therapy for HAT. In areas where Elisa HIV tests are not readily available, repeating the HIV RDT at the end of therapy may help to identify roughly half of false positives.

## Introduction

En 2019 selon l'OMS, des cas de maladie du sommeil ou trypanosomiase humaine africaine (THA) ont encore été rapportés dans 12 pays d'Afrique sub-saharienne. En raison de la forte prévalence des infections VIH dans certains de ces pays, la faible spécificité de certains tests de diagnostic rapide (TDR) VIH chez des patients souffrant de THA a été remarquée [1, 3]. Il a également été établi que chez les patients avec une THA, qui ont un TDR VIH initial faussement positif, celui-ci se négative plusieurs mois après le traitement de la THA, mais il n'existe pas de données plus précises sur la cinétique de ce phénomène. Comprendre les mécanismes sur lesquels reposent ces TDR VIH faussement positifs peut contribuer à augmenter la précision du dépistage du VIH, principalement dans les zones endémiques pour la THA. Nous rapportons le cas d'une patiente prise en charge comme une méningite à cryptocoque opportuniste d'un sida, qui avait en fait une THA. Les résultats d'une étude que nous avons menée ultérieurement sur une cohorte rétrospective de patients présentant une THA, apportent des informations nouvelles sur la cinétique de négativation du TDR VIH faussement positif, consécutivement au traitement de la THA.

## Matériel et Méthodes

### Cas clinique

En août 2016, une patiente de 21 ans est admise aux urgences de Limoges pour confusion. Elle arrive de République démocratique du Congo en France en avril 2016. Elle est fébrile à 40 °C, léthargique, sans raideur de nuque ni signe neurologique de focalisation. Le bilan biologique ne montre qu'une thrombopénie (113109 plaquettes/litre), et une CRP à 1 mg/l. La recherche de paludisme est négative. Un TDR VIH (Determine HIV 1/2) est positif. Une cryptococcose neuro-méningée est suspectée et une ponction lombaire est réalisée aux urgences. L'analyse du liquide cérébrospinal (LCS) trouve 225 leucocytes/mm^3^ (65% de lymphocytes, 33% de cellules indifférenciées, et 2% de polynucléaires neutrophiles), la glycorachie est normale et la protéinorachie à 0,68 g/l. Le test à l'encre de Chine pour la recherche de *Cryptococcus neoformans* sur le LCS est négatif, mais met en évidence la présence de trypomastigotes de *Trypanosoma brucei* permettant de faire le diagnostic de THA au stade 2. Le test de trypanolyse immunitaire contre le variant de surface glycoprotéine LiTat 1.3 et 1.5 (Institut de médecine tropicale d'Anvers, Belgique) est positif sur un échantillon de sérum, précisant que la patiente est atteinte par *Trypanosoma brucei gambiense* de type I. Le test de confirmation ELISA pour le VIH est négatif.

### Étude rétrospective de cohorte

Les données ont été collectées parmi une cohorte de 253 patients diagnostiqués avec une THA à *Trypanosoma brucei gambiense*. Cette cohorte a été constituée à l'occasion d'une étude, financée par la Foundation for Innovative and New Diagnostic (FIND), menée en Angola entre 2008 et 2011 dans les provinces de Uíge, Bengo et Kwanza Norte après screening de l'ensemble de la population à partir de l'âge de 12 ans. Les patients déclarés positifs ont été revus en fin de traitement, puis à 6, 12, 18 et 24 mois. Après aliquotage, les prélèvements ont été conservés à -80 °C. La constitution de cette cohorte a été validée par le comité d'éthique dépendant de la « Direccao National de Saude » d'Angola. Le comité consultatif sur le traitement de l'information en matière de recherche a émis un avis favorable sous la référence 08.228bis.

Le statut VIH initial avait été déterminé avec un TDR VIH de type Vikia (Biomérieux, France). Pendant les deux premières semaines, les patients au stade 1 ont été traités par pentamidine et les patients au stade 2 par eflornithine (DFMO).

Les patients étaient examinés au moment du diagnostic, puis à J14 (uniquement ceux au stade 2). Pour tous les patients inclus, le suivi des prélèvements était organisé à J14 puis tous les 6 mois pendant 18 mois après le traitement [2, 4]. Les prélèvements initiaux stockés ont été repris rétrospectivement pour déterminer le nombre de faux positifs en TDR VIH (Vikia HIV1/2, Biomérieux, France) en réalisant un test ELISA et un Western Blot.

Chez les patients dont le TDR VIH était initialement faussement positif, les prélèvements suivants étaient repris pour réaliser un TDR VIH (Vikia HIV1/2, Biomérieux, France), et des tests ELISA et Western Blot.

## Résultats et Discussion

Parmi les 253 patients de la cohorte angolaise, 21 avaient un TDR VIH positif. Sur ces 21 patients, 86% (18/21) se sont révélés de faux positifs après réalisation d'un test ELISA et Western Blot négatifs. Ces 18 patients étaient au stade 2 de la maladie et ils ont été traités par DFMO.

**Figure 1:**
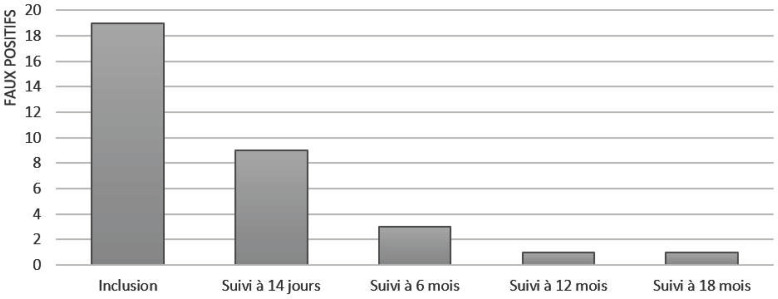
Nombre de faux positifs (VIKIA HIV 1/2®, BioMérieux) en fonction du temps après le traitement trypanocide Number of false positives HIV rapid diagnostic tests (VIKIA HIV 1/2®, BioMérieux) during follow-up

Au cours du suivi, parmi les 18 patients avec un test HIV faussement positif, 50% (9/18) avaient un TDR HIV négatif à J14, 84% (16/18) à 6 mois, 94% (17/18) à 12 mois et 94% (17/18) à 18 mois (Fig. 1).

Notre étude apporte de nouvelles données sur la clairance de tests VIH faussement positifs chez des patients atteints de THA puisque la moitié des TDR VIH faussement positifs étaient négativés après 2 semaines de traitement.

Dans les zones endémiques de THA, les tests Elisa VIH ne sont pas toujours disponibles immédiatement. Répéter le TDR VIH en fin de traitement de la THA (au 14ème jour) peut s'avérer utile pour identifier les faux positifs de TDR VIH. Cela ne s'applique peut-être pas de la même façon à tous les types de TDR VIH et d'autres études seraient nécessaires pour préciser ce point.

La négativation, inhomogène dans le temps, de ces faux positifs chez des patients porteurs de THA suggère la présence de plusieurs mécanismes pouvant l'expliquer. En l'absence de données sur la parasitémie initiale, un rôle direct de la biomasse du parasite comme cause de fausse positivité de TDR VIH ne peut être écarté (un seuil de parasitémie au-dessus duquel le TDR VIH se positiverait). Cependant, un rôle direct de la parasitémie dans la fausse positivité des TDR VIH conduirait à une proportion élevée de TDR VIH faussement positifs chez les patients présentant une THA, ce qui n'est pas le cas. De plus, la THA n'est pas la seule infection qui entraîne de faux positifs en TDR VIH; c'est le cas également pour la leishmaniose et le paludisme.

Une explication plus plausible de ces TDR VIH faussement positifs repose sur une réactivité croisée entre des antigènes du trypanosome et ceux du virus du VIH, en lien avec une forte activation polyclonale B au début de la THA [1]. L'hétérogénéité de la cinétique de négativation des TDR HIV pourrait refléter une hétérogénéité de clairance des anticorps produits par l'activation polyclonale B, avec une clairance plutôt rapide d'anticorps impliqués dans la réaction croisée. Les déterminants parasitaires impliqués dans la fausse positivité des TDR VIH restent à préciser.

Le cas clinique alerte sur le risque de faux diagnostic d'infection par le VIH, quand le diagnostic ne repose que sur la base des résultats d'un TDR VIH [1], particulièrement chez les patients atteints de THA à *Trypanosoma brucei gambiense*. Enfin, le diagnostic de THA doit être évoqué chez tout patient revenant de zone d'endémie avec un tableau de méningoencéphalite non étiquetée.

## Conclusion

Alors que les TDR VIH faussement positifs chez des patients au stade 2 de la THA sont assez fréquents, nous montrons pour la première fois que la moitié d'entre eux a disparu à la fin du traitement de la THA. Ces données suggèrent une clairance précoce de certains anticorps impliqués dans la réaction croisée entre le VIH et la THA.

Dans les régions où les tests VIH Elisa ne peuvent être réalisés rapidement, répéter le TDR VIH à la fin du traitement de la THA permettrait d'identifier la moitié des TDR VIH faussement positifs.

## Remerciements

Philippe Büscher, Unit of Parasite Diagnostics, Department of Biomedical Sciences, Institute of Tropical Medicine, Nationalestraat 155, 2000 Antwerpen, Belgium

Marie-Laure Dardé, Laboratory of Mycology and Parasitology, CHU Dupuytren, Limoges, France

## Conflits D'intérêts

Les auteurs ne déclarent aucun conflit d'intérêt.
